# Arbuscular mycorrhizal fungi increase salt tolerance of apple seedlings

**DOI:** 10.1186/s40529-014-0070-6

**Published:** 2014-10-09

**Authors:** Shou-Jun Yang, Zhong-Lan Zhang, Yuan-Xia Xue, Zhi-Fen Zhang, Shu-Yi Shi

**Affiliations:** College of Science and Technology, Yantai Institute, China Agricultural University, Binhai Middle Road 2006, Yantai, 264670 China

**Keywords:** Arbuscular mycorrhizal fungi, Inoculation, Salinity, Apple seedling, Physiological characteristic

## Abstract

**Background:**

Apple trees are often subject to severe salt stress in China as well as in the world that results in significant loss of apple production. Therefore this study was carried out to evaluate the response of apple seedlings inoculated with abuscular mycorrhizal fungi under 0, 2‰, 4‰ and 6‰ salinity stress levels and further to conclude the upper threshold of mycorrhizal salinity tolerance.

**Results:**

The results shows that abuscular mycorrhizal fungi significantly increased the root length colonization of mycorrhizal apple plants with exposure time period to 0, 2‰ and 4‰ salinity levels as compared to non-mycorrhizal plants, however, percent root colonization reduced as saline stress increased. Salinity levels were found to negatively correlate with leaf relative turgidity, osmotic potential irrespective of non-mycorrhizal and mycorrhizal apple plants, but the decreased mycorrhizal leaf turgidity maintained relative normal values at 2‰ and 4‰ salt concentrations. Under salt stress condition, Cl^−^ and Na^+^ concentrations clearly increased and K^+^ contents obviously decreased in non-mycorrhizal roots in comparison to mycorrhizal plants, this caused mycorrhizal plants had a relatively higher K^+^/Na^+^ ratio in root. In contrast to zero salinity level, although ascorbate peroxidase and catalase activities in non-inoculated and inoculated leaf improved under all saline levels, the extent of which these enzymes increased was greater in mycorrhizal than in non-mycorrhizal plants. The numbers of survived tree with non-mycorrhization were 40, 20 and 0 (i.e., 66.7%, 33.3% and 0) on the days of 30, 60 and 90 under 4‰ salinity, similarly in mycorrhization under 6‰ salinity 40, 30 and 0 (i.e., 66.7%, 50% and 0) respectively.

**Conclusion:**

These results suggest that 2‰ and 4‰ salt concentrations may be the upper thresholds of salinity tolerance in non-mycorrhizal and mycorrhizal apple plants, respectively.

**Electronic supplementary material:**

The online version of this article (doi:10.1186/s40529-014-0070-6) contains supplementary material, which is available to authorized users.

## Background

Salinity is considered as one of the most important abiotic stresses that limits crop productivity, affecting several aspects of plant metabolism that generally results in the reduction of plant growth in non-halophytes plants (Chinnusamy *et al*. [2005]; Flowers [2004]; Munns and Termaat [1986]). Generally, salinity effects are the combined result of the complex interaction among different morphological, physiological, and biochemical processes (Singh and Chatrath [2001]). Under salt stress condition, plants are often stressed in three ways: (1) low water potential in the root medium leads to water deficits in plants, (2) the toxic effects of ions, mainly Na and Cl, and (3) nutrient imbalance caused by depression in uptake and/or transport (Marschner [1995]). In addition, production of reactive oxygen species is also a major damaging factor in plants exposed to salinity stress (Aggarwal *et al*. [2012]).

Apple (*Malus* × *domestica* Borkh*.*) is one of the most valuable horticultural fruit crops in the world. Although it produces the highest yield in China, it is not a halophyte species and sensitive to salinity, therefore it is subject to severe salt stress in many areas (Flowers [1999]; Xia *et al*. [2005]; Navarro [2003]). To deal with saline soil and minimize apple loss, many strategies were proposed to counteract salt detrimental effects such as searching for new salt-tolerant apple rootstock, removing excessive salt accumulation in groundwater and desalinizing water for irrigation (Reza *et al*. [2012]; Bouksilaa *et al*. [2013]). Though successful, these approaches are costly and out of reach for developing countries. As a result, such strategies are obviously limited in a short term period and also not achievable with stationary apple plants. Up to now, the loss of apple productivity caused by salt stress is still a major concern.

Abuscular mycorrhizal (AM) fungi are associated with the roots of over 90% of terrestrial plant species (Gadkar *et al*. [2001]). Many investigations shows that AM symbiosis contributes to plant growth and nutrient uptake, improve fruit quality and enhance several abiotic stresses such as low temperature stress, drought, salt stress, etc. (Azcon-Aguilar and Barea [1997]; Mena-Violante *et al*. [2006]; Miransari [2010]). Munns ([1993]) demonstrated that plant growth in saline medium was affected first by an osmotic stress and then by toxic and nutritive stresses. Coincidentally, mycorrhizal colonization is able to compensate such disequilibria (Ruiz-Lozano [2003]; Marulanda *et al*. [2006]). AM colonization is capable of increasing resistance of the host plant to salinity is also induced by other mechanisms. Several studies suggest that AM symbiosis helps plants to alleviate salt stress by enhancing the activities of antioxidant enzymes (Alguacil *et al*. [2003]; Harisnaut *et al*. [2003]; He *et al*. [2007]). Although AM fungi can increase host plants to resist salt stress, the capabilities of which depend on the behavior of each species. Porras-Soriano *et al*. ([2009]) tested the efficacy of three species of AM fungi – *Glomus mosseae*, *G. intraradices* and *G. claroideum* – to alleviate salt stress in olive trees under nursery conditions, who observed that *G. mosseae* was the most efficient fungus in terms of olive tree performance and particularly in the protection offered against the detrimental effects of salinity.

At present, few researches focus on mechanisms in mycorrhizal apple plants to alleviate salt stress. Therefore, this study is undertaken to identify the potential interaction between AM fungi and apple plants in salinity environment and further to define the upper threshold of mycorrhizal salinity tolerance of apple seedlings.

## Methods

### Plant material and AM inoculum

Experimental plants were one-year-old Red Fuji apple seedlings (*Malus hupehensis* Rehd. root stock) with trunk diameter of 0.7 cm and height of 50 cm. The selected mycorrhizal inoculum of *Glomus versiforme* consisted of 15 isolated spores per milliliter and was provided by Qingdao Agricultural University, Shandong Province, China. It was derived from pot culture prepared with *Trifolium repens* L. grown in 1:9 sterilized soil-sand and contained colonized pieces of root, soil and spores.

### Experimental design

The experimental design consisted of four treatments with four salinity levels (NaCl: 0, 2‰, 4‰ and 6‰), each treatment involved in 10 non-AM inoculated and 10 AM inoculated plants and replicated six times. Apple seedlings were transplanted in plastic pots (20 cm in diameter and 0.05 m^3^ in volume), each filled with 25 kg of brawn soil sterilized at 121°C for 2‰hours and its chemical properties were as followings: organic matter 7.64‰g kg^−1^, available nitrogen 24.36‰mg kg^−1^, available phosphorus 3.17 mg kg^−1^, available potassium 61.24‰mg kg^−1^, salinity 0.093‰ and pH 7.2 (1: 2.5, soil: water suspension). In the transplantation process, in order to assure a rapid colonization the root systems of AM treatment were uniformly sprinkled with 100 ml *Glomus versiforme* inoculum. Counterparts of non-AM treatment were received volumetric sterilized soil-sand free of spores. After apple seedlings survived, the pots were watered with 1000 ml saline solution every 15 days throughout the experiment, fresh water was added as necessary to make up for losses by evaporation or transpiration. Determination was conducted at 30, 60 and 90 days after salt treatment.

### Data collection and determination method

Root length colonization by AM fungi was calculated using a gridline intersect method after staining the roots with trypan blue (Koske and Gemma [1989]). Leaf protein extraction was according to the method of Blilou *et al*. ([2000]) and protein concentration was determined as described by Bradford ([1976]). Catalase and ascorbate peroxidase activities in leaf were measured as described by Barber ([1980]) respectively. The cations K^+^ and Na^+^ concentrations in root were analyzed using atomic absorption spectroscopy and Cl^−^ concentration in root was determined by the method of AgNO_3_ titration (Lu [1999]). The osmotic potential was determined by the method of Gusov ([1960]). Leaf relative turgidity was estimated according to the following equation (Nomir [1994]).

$$\mathrm{Leaf}\;\mathrm{relative}\;\mathrm{turgidity}=\frac{\mathrm{Fresh}\;\mathrm{weight}\;-\;\mathrm{Dry}\;\mathrm{weight}}{\mathrm{Turgid}\;\mathrm{weight}\;-\;\mathrm{Dry}\;\mathrm{weight}}$$

### Statistical analysis

All experiments were repeated as indicated. Values presented are means. The effects of the treatments were tested by one-way analysis of variance (ANOVA). Means were compared between the treatments using the *LSD* (least significant difference) test at the 0.05 probability level.

## Results

### Effect of different salinity levels on root length colonization of apple seedlings inoculated and non-inoculated with AM fungi

In non-mycorrhizal plants root length colonization rate was always zero, however, mycorrhizal plants presented a trend in increase in percent root colonization at zero, low (2‰), moderate (4‰) and high (6‰) salinity levels. Table 1 demonstrates that the maximum value of root length colonization occurred in mycorrhizal plants under no saline condition and it clearly decreased under salinity stress environment throughout the experiment. In mycorrhizal plants root colonization percentage continually increased with time period at zero, low and moderate salinity levels except for that root colonization rate gradually reduced with prolonged exposure to high salinity level. With an increasing salinity stress level, percent AM colonization of mycorrhizal plants significantly reduced, especially at 90 days of high salinity stress level AM inoculation root length colonization could not be tested due to apple plants were all dead.

**Table 1:** **Effect of different salinity levels on root length colonization of apple seedlings inoculated and non-inoculated with** ***Glomus versiforme***

Treatments	Root length colonization (%)					
	Non-inoculation			Inoculation		
	30	60	90	30	60	90
0	0	0	0	28.1^a^	44.3^a^	56.9^a^
2‰	0	0	0	20.7^b^	35.4^b^	46.2^b^
4‰	0	0	×	19.2^c^	27.7^c^	35.6^c^
6‰	0	×	×	18.2^c^	10.8^d^	×

^a^Values of each column followed by the same letter are not significantly different at the 0.05 level. Each value represents the mean of 6 replicates. Numerals 30, 60 and 90 stand for sampling times (days) after salinity treatment. The sign “×” denotes that apple plants were dead.

### Changes of leaf relative turgidity and osmotic potential inoculated and non-inoculated with AM fungi under different salinity levels

**Figure 1:**
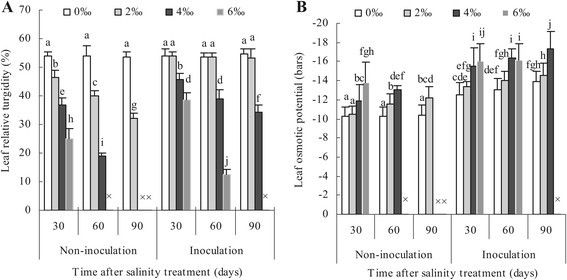
**Relative turgidity (A) and osmotic potential (B) of apple leaves inoculated and non-inoculated with** ***Glomus versiforme*** **.** Each bar represents a mean ± standard deviations. Values sharing the same letter are not significantly different at the 0.05 level. Each value represents the mean of 6 replicates. The sign “×” denotes that apple plants were dead.

Under no saline condition, leaf relative turgidities in non-mycorrhizal and mycorrhizal plants remained a comparative steady-state level from 53.75% to 54.56% throughout the experiment (Figure 1A). As salinity stress was fulfilled, the significant decreases in leaf relative turgidities occurred in non-mycorrhizal and mycorrhizal plants at all salinity levels and only at moderate and high salt levels, respectively. At zero salt stress level, no deviations were seen in leaf osmotic potential of non-mycorrhizal plants in the whole trial, however in mycorrhizal plants leaf osmotic potential obviously decreased (Figure 1B). Irrespective of non-inoculated and inoculated plants, leaf osmotic potentials all decreased under low salinity stress condition. With an increasing salinity level and day of salinity treatment, the decrease rate of leaf osmotic potential in mycorrhizal plants was higher than in non-mycorrhizal plants, especially on the 60 days of high salt stress level mycorrhizal leaf osmotic potential still remained a relative lower value of −16.11 bars but non-mycorrhizal plants were dead.

### Ascorbate peroxidase and catalase activities of mycorrhizal apple leaf affected by different salinity levels

**Figure 2:**
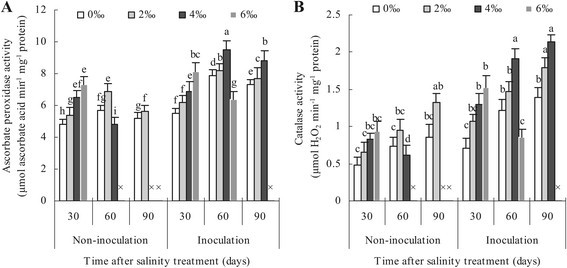
**Ascorbate peroxidase activity (A) and catalase activity (B) of apple leaves inoculated and non-inoculated with** ***Glomus versiforme*** **.** Each bar represents a mean ± standard deviations. Each value represents the mean of 6 replicates. The sign “×” denotes that apple plants were dead.

There were similar trends between non-mycorrhizal and mycorrhizal plants in increase in ascorbate peroxidase and catalase activities under different salt stress levels, nevertheless the increase rate was higher in mycorrhizal plants as compared to non-mycorrhizal plants (Figure 2A, B). The peak values of ascorbate peroxidase activity always appeared on the 60 days and subsequently decreased in non-mycorrhizal plants at zero, low salt levels and in mycorrhizal plants at zero, moderate and high salt levels. Unlike ascorbate peroxidase, the values of catalase activity gradually increased and the maximums occurred on the 90 days with respect to all salt stress levels. Figure 2 also showed the responses of ascorbate peroxidase and catalase activities in non-mycorrhizal plants stressed by moderate salinity were similar to those in mycorrhizal plants at high salinity stress level.

### Effect of salinity stress on the concentrations of Cl-, K+, Na+ and the ratio of K+ to Na+ in non-mycorrhhizal and mycorrhizal apple root

Table 2 indicates that salinity stress resulted in increases in the concentrations of Cl^-^ and Na^+^ in plant root, regardless of non-mycorrhizal and mycorrhizal plants, but the extent of which they raised was diverse. When apple trees were stressed under zero salt level, the Cl^-^ and Na^+^ contents in non-mycorrhhizal and mycorrhizal roots ranged from about 0.08 to 0.09 g kg^-1^ at 30 and 60 days, but they increased to 0.1272 and 0.093 g kg^-1^ at 90 days, respectively. With an increasing salinity stress level and exposure time period to salinity stress, the increase rates of Cl^-^ and Na^+^ concentration in non-mycorrhhizal plants were apparently higher than in mycorrhizal plants. Compared to non-mycorrhizal plants, K^+^ concentrations in mycorrhizal roots slightly increased under zero salt level and were obviously higher under moderate and high salinity stress. Due to lower Na^+^ concentration and higher K^+^ concentration in mycorrhizal roots in comparison to non-mycorrhhizal roots, therefore ratios of K^+^ to Na^+^ ions in roots were higher than in non-mycorrhizal plants.

**Table 2:** **Effect of different salinity levels on the concentrations of Cl** ^**-**^ **, K** ^**+**^ **, Na** ^**+**^ **and the ratio of K** ^**+**^ **to Na** ^**+**^ **in apple roots inoculated and non-inoculated with** ***Glomus versiforme***

Treatment	Non-inoculation					Inoculation			
		Cl^-^(g kg^-1^)	K^+^(g kg^-1^)	Na^+^(g kg^-1^)	K^+^/Na^+^	Cl^-^(g kg^-1^)	K^+^(g kg^-1^)	Na^+^(g kg^-1^)	K^+^/Na^+^
0	30	0.0872^f^	1.529^d^	0.0565^f^	27.062^a^	0.0809^e^	1.533^f^	0.0533^e^	28.77^a^
	60	0.0982^f^	1.720^bc^	0.0636^f^	27.044^a^	0.0856^e^	1.777^f^	0.0612^de^	29.02^a^
	90	0.1272^e^	2.060^a^	0.0824^e^	25.000^b^	0.0933^e^	2.130^e^	0.0795^d^	26.79^b^
2‰	30	0.2919^d^	1.393^e^	0.1891^d^	7.366^c^	0.1713^d^	3.150^d^	0.1110^c^	28.38^a^
	60	0.3911^c^	1.606^cd^	0.2534^c^	6.338^d^	0.2249^c^	4.173^bc^	0.1457^c^	28.64^a^
	90	0.5126^b^	1.870^b^	0.3321^b^	5.631^e^	0.3428^b^	5.795^a^	0.2221^b^	26.09^b^
4‰	30	0.3459^c^	1.154^f^	0.2241^c^	5.149^e^	0.2357^c^	3.483^cd^	0.1527	22.81^c^
	60	0.5581^ab^	1.304^e^	0.3616^ab^	3.606^f^	0.3856^b^	5.181^ab^	0.2498^ab^	20.74^d^
	90	×	×	×	×	0.4820^a^	5.325^a^	0.3123^a^	17.05^e^
6‰	30	0.6013^a^	1.071^f^	0.3896^a^	2.749^j^	0.3535^b^	3.774^c^	0.2290^b^	16.48^e^
	60	×	×	×	×	0.5035^a^	4.521^b^	0.3262^a^	13.86^f^
	90	×	×	×	×	×	×	×	×

^a^Values of each column followed by the same letter are not significantly different at the 0.05 level. Each value represents the mean of 6 replicates. Numerals 30, 60 and 90 stand for sampling times (days) after salinity treatment. The sign “×” denotes that apple plants were dead.

### The number of survived apple seedlings inoculated and non-inoculated with AM fungi under different salinity levels

**Figure 3:**
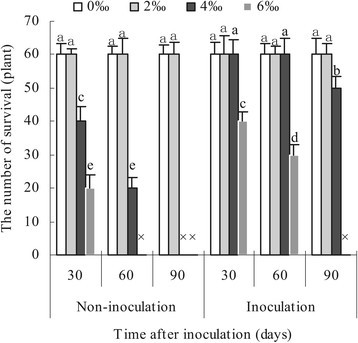
**The number of survival of apple seedlings inoculated and non-inoculated with** ***Glomus versiforme*** **.** Each bar represents a mean ± standard deviations. Values sharing the same letter are not significantly different at the 0.05 level. Each value represents the mean of 6 replicates. The sign “×” denotes that apple plants were dead.

Results in Figure 3 shows that whether AM fungi were inoculated or not 100% apple seedlings survived under salinity stress from zero to low salt concentrations from 30 to 90 days after salt treatment. With the use of moderate saline solution, the numbers of survival were 40, 20, 0 (i.e., 66.7%, 33.3%, 0) in non-mycorrhization apple seedling and 60, 60, 50 (i.e., 100%, 100%, 83.3%) in mycorrhization apple seedlings after 30, 60 and 90 days of salinity stress, respectively. When salinity stress was set as high salt concentration, survival durations were only 30 days in 20 non-mycorrhizal seedlings and 60 days in 30 mycorrhizal seedlings, respectively. Data suggested that the number of survived apple seedlings responded negatively to salt concentration and exposure time to salinity stress.

## Discussion

The finding that AM fungi inoculation with apple seedlings could improve salt tolerance of host plants under stress condition is very important information for saline areas in China as well as in the world. These results evidence that, to reduce the unfavorable effects of salinity on apple plant growth, the use of AM inoculation ought to be considered as biological method to alleviate salinity stress.

Root length colonization rate is an important indicator suggesting plants become mycorrhizal. In the present study root colonization rate was found to negatively correlate with salinity stress. This conclusion is consistent with previous report that soil salinity can affect AM fungi by slowing down root colonization, spore germination (Juniper and Abbott [1993]). However, other studies showed that there is in fact no reduction in AM colonization in the presence of NaCl and even increases in sporulation and colonization occur (Aliasgharzadeh *et al*. [2001]; Yamato *et al*. [2008]; Peng *et al*. [2010]). The discrepancies amongst studies may be because that various AM fungal spp. have varying tolerance to salinity (Porras-Soriano *et al*. [2009]).

Plants in saline soils are subjected to physiological drought as Na^+^ and Cl^-^ ions bind water that is needed to be mobilized by the plants (Fuzy *et al*. [2008]). In the presence of salinity stress, mycorrhizal apple seedlings maintained relatively higher leaf turgidity compared to non-mycorrhizal plants. The phenomenon is ascribed to improved hydraulic conductivity of apple plants with a longer root and an altered root system morphology induced by AM fungi (Dehne [1982]; Kothari *et al*. [1990]). In our study apple seedlings inoculated with AM fungi were shown to possess a lower leaf osmotic potential in contrast to non-inoculated plants under salt stress condition. The result might be attributed to the reason that fungal hyphae extend from the root surface into the soil, increase the surface areas of root and thus acquire more macroelement beyond the depletion zone (Schnepf *et al*. [2011]). The fungal accumulating solutes maintain a relatively lower osmotic potential beneficial for plant osmotic adjustment, consequently enabling host plants to use water more efficiently (Graham and Syversten [1984]).

There is accumulating evidence that production of reactive oxygen species is a major damaging factor in plants exposed to different environmental stresses, including salinity (Hernandez *et al*. [1995]). Results shows that an increase in leaf ascorbate peroxidase and catalase activities was under low salinity and a decrease was under high salinity in non-mycorrhizal and mycorrhizal apple plants, however, mycorrhizal plants demonstrated higher activity than non-mycorrhizal plants, meaning that AM fungi induced more effective defense mechanisms to protect host from detrimental effects of salt stress.

Under salt stress environment, roots of non-mycorrhizal and mycorrhizal apple plants were forced to take up more Cl^-^ and Na^+^, because NaCl was used to develop a salinity gradient. Root Cl^-^ and Na^+^ concentrations were lower in mycorrhizal than in non-mycorrhizal plants under given salinity conditions, resulting from dilution effects due to growth enhancement by AM fungi colonization (Al-Karaki, [2000]). In this study, root concentration of K^+^ was higher for mycorrhizal than for non-mycorrhizal seedlings at all salinity levels. It seems that improved plant nutrition by AM symbiosis allows cells to regulate and separate flowing ions more effectively (Giri *et al*. [2007]). The nutrient imbalance due to salt stress results from the effects of salinity on nutrient availability, competitive uptake, transport, or partitioning within the plants (Grattan and Grieve [1993]). The results here that mycorrhizal roots kept a higher K^+^/Na^+^ ratio confirmed that K^+^ competed for the site of Na^+^ on the cell membrane. The conclusion was also reported by Hu and Schmidhalter ([2005]) Maintenance of a high cytosolic K^+^/Na^+^ ratio is a key feature of plant salt tolerance (Cuin *et al*. [2008]). The competition of K^+^ due to mycorrhization may induce the decrease of Na^+^, thus enhancing salt tolerance of mycorrhizal apple plants.

The data in survived apple seedlings verifies that 4‰ and 6‰ salt concentration were the limits of salinity tolerance in non-mycorrhizal and mycorrhizal apple seedlings, respectively. This further confirmed that AM fungi increased salinity tolerance of mycorrhizal apple seedlings as compared to non-mycorrhizal counterparts exposed to salinity stress.

## Conclusion

In conclusion, non-mycorrhizal and mycorrhizal apple seedlings existed similar performances with respect to salinity stress such as reduced leaf turgidity, osmotic potential, K^+^/Na^+^ ratio, the number of survival and induced antioxidant enzymes, but the extents of which they responded to salinity stress were not the completely same. 2‰ and 4‰ salt concentrations may be the upper thresholds of salinity tolerance in non-mycorrhizal and mycorrhizal apple plants, respectively.

## Authors’ original submitted files for images

Below are the links to the authors’ original submitted files for images. Authors’ original file for figure 1 Authors’ original file for figure 2 Authors’ original file for figure 3

## Abbreviation

AM: Abuscular mycorrhizal

## References

[1] Aggarwal A, Kadian N, Karishma N, Tanwar A, Gupta KK (2012). Arbuscular mycorrhizal symbiosi and alleviation of salinity stress. J Appl Nat Sci.

[2] Alguacil MM, Hernandez JA, Caravaca F, Portillo B, Roldan A (2003). Antioxidant enzyme activities in shoots from three mycorrhizal shrub species afforested in a degraded semi-arid soil. Plant Physiol.

[3] Aliasgharzadeh N, Saleh RN, Towfighi H, Alizadeh A (2001). Occurrence of arbuscular mycorrhizal fungi in saline soils of the Tabriz Plain of Iran in relation to some physical and chemical properties of soil. Mycorrhiza.

[4] Al-Karaki GN (2000). Growth of mycorrhizal tomatoand mineral acquistion under salt stress. Mycorrhiza.

[5] Azcon-Aguilar C, Barea JM (1997). Applying mycorrhiza biotechnology to horticulture: significance and potentials. Hort Sci.

[6] Barber JM (1980). Catalase and peroxidase in primary leaves during development and senescence. Z Pflanzenphysiol.

[7] Blilou I, Bueno P, Ocampo JA, García-Garrido JM (2000). Induction of catalase and ascorbate peroxidase activities in tobacco roots inoculated with the arbuscular mycorrhizal Glomus mosseae. Mycol Res.

[8] Bouksilaa F, Bahrib A, Berndtssonc R, Perssond M, Rozemae J, Van der Zeef SEATM (2013). Assessment of soil salinization risks under irrigation with brackish water in emiarid Tunisia. Environ Exp Bo.

[9] Bradford MM (1976). A rapid and sensitive method for the quantitation of microgram quantities of protein utilizing the principle of protein-dye binding. Anal Biochem.

[10] Chinnusamy V, Jagendorf A, Zhu JK (2005). Understanding and different types of soil stress. Plant Biol.

[11] Cuin TA, Betts SA, Chalmandrier R, Shabala S (2008). A root’s ability to retain K^+^ correlates with salt tolerance in wheat. J Exp Bot.

[12] Dehne HW (1982). Interaction between vesicular-arbuscular mycorrhizal fungi and plant pathogens. Phytopathology.

[13] Flowers TJ (1999). Salinisation and horticultural production. Hort Sci.

[14] Flowers TJ (2004). Improving crop salt tolerance. J Exp Bot.

[15] Fuzy A, Biro B, Toth T, Hildebrandt U, Bothe H (2008). Drought, but not salinity, determines the apparent effectiveness of halophytes colonized by arbuscular mycorrhizal fungi. Plant Physiol.

[16] Gadkar V, David-Schwartz R, Kunik T, Kapulnik Y (2001). Arbuscular mycorrhizal fungal colonization. Factors involved in host recognition. Plant Physiol.

[17] Giri B, Kapoor R, Mukerji KG (2007). Improved tolerance of *Acacia nilotica* to salt stress by arbuscular mycorrhiza, *Glomus fasciculatum* may be partly related to elevated K/Na ratios in root and shoot tissues. Microb Ecol.

[18] Graham JH, Syversten JP (1984). Influence of vesicular arbuscular mycorrhiza on the hydraulic conductivity of roots of two Citrus rootstocks. New Phytol.

[19] Grattan SR, Grieve CM, Pessarakli M (1993). Mineral Nutrient Acquisition and Response by Plants Grown in Saline Environments. Handbook of Plant and Crop Stress.

[20] Gusov NA (1960). Some Methods in Studying Plant Water Relations.

[21] Harisnaut P, Poonsopa D, Roengmongkol K, Charoensataporn R (2003). Salinity effects on antioxidant enzymes in mulberry cultivar. Sci Asia.

[22] He ZQ, He CX, Zhang ZB, Zou ZR, Wang HS (2007). Changes of antioxidative enzymes and cell membrane osmosis in tomato colonized by arbuscular mycorrhizae under NaCl stress. Collo Surf B Biointer.

[23] Hernandez JA, Olmos E, Corpas GJ, Sevilla F, del Rio LA (1995). Salt induced oxidative stress in chloroplast of pea plants. Plant Sci.

[24] Hu Y, Schmidhalter U (2005). Drought and salinity: a comparison of their effects on mineral nutrition of plants. J Plant Nutr Soil Sci.

[25] Juniper S, Abbott L (1993). Vesicular-arbuscular mycorrhizas and soil salinity. Mycorrhiza.

[26] Koske RE, Gemma JN (1989). A modified procedure for staining roots to detect VA mycorrhizae. Mycol Res.

[27] Kothari SK, Marschner H, George E (1990). Effect of VA mycorrhizal fungi and rhizosphere microorganism on root and shoot morphology, growth and water relations of maize. New Phytol.

[28] Lu RK (1999). Analytical Methods for Soil and Agro-Chemistry.

[29] Marschner H (1995). Mineral Nutrition of Higher Plants.

[30] Marulanda A, Barea JM, Azćon R (2006). An indigenous droughttolerant strain of Glomus intraradices associated with a native bacterium improves water transport and root development in Retama sphaerocarpa. Microb Ecol.

[31] Mena-Violante HG, Ocampo-Jimenez O, Dendooven L, Martinez-Soto G, Gonzalez- Castaneda J, Davies FT, Olalde-Portugal V (2006). Arbuscular mycorrhizal fungi enhanced fruit growth and quality of chile ancho (Capsicum annuum L. cv San Luis) plants exposed to drought. Mycorrhiza.

[32] Miransari M (2010). Contribution of arbuscular mycorrhizal symbiosis to plant growth under different types of soil stress. Plant Biol.

[33] Munns R (1993). Physiological processes limiting plant growth in saline soil: some dogmas and hypothesis. Plant Cell Environ.

[34] Munns R, Termaat A (1986). Whole plant response to salinity. Aust J Plant Physiol.

[35] Navarro JM, Garrido C, Martínez V, Carvajal M (2003). Water relations and xylem transport of nutrients in pepper plants grown under two different salts stress. Plant Growth Regul.

[36] Nomir SA (1994). Physiological Studies on Kaki.

[37] Peng J, Li Y, Shi P, Chen X, Lin H, Zhao B (2010). The differential behavior of arbuscular mycorrhizal fungi in interaction with *Astragalus sinicus* L. under salt stress. Mycorrhiza.

[38] Porras-Soriano A, Soriano-Martín ML, Porras-Piedra A, Azcón R (2009). Arbuscular mycorrhizal fungi increased growth, nutrient uptake and tolerance to salinity in olive trees under nursery conditions. Plant Physiol.

[39] Reza B, Mansour G, Ali-Akbar M, Raof A (2012). Effects of salinity on *In vitro* shoot proliferation and rooting of apple rootstock MM.106. World Appl Sci J.

[40] Ruiz-Lozano JM (2003). Arbuscular mycorrhizal symbiosis and alleviation of osmotic stress: new perspectives for molecular studies. Mycorrhiza.

[41] Schnepf A, Jones D, Roose T (2011). Modelling nutrient uptake by individual hyphae of arbuscular mycorrhizal fungi: temporal and spatial scales for an experimental design. Bull Math Biol.

[42] Singh KN, Chatrath R, Reynolds MP, Monasterio JIO, McNab A (2001). Salinity Tolerance. Application of Physiology in Wheat Breeding.

[43] Xia Y, Huimin H, Shu HR, Wang TM, Liu DX, Fang YF, Chai CH (2005). Changes of leaf membrane penetration, proline and mineral nutrient contents of young apple tree under NaCl stress. Fruits.

[44] Yamato M, Ikeda S, Iwase K (2008). Community of arbuscular mycorrhizal fungi in a coastal vegetation on Okinawa island and effect of the isolated fungi on growth of sorghum under salt-treated conditions. Mycorrhiza.

