# Supplementary figures and images for: Arbuscular mycorrhizal fungi increase salt tolerance of apple seedlings
Source: Bot Stud. 2014 Oct 9;55:70. doi: 10.1186/s40529-014-0070-6 (PMC5430355; doi:10.1186/s40529-014-0070-6)

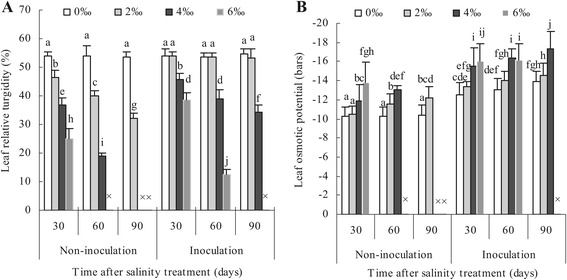

Supplement: Supplementary file 1 — Authors’ original file for figure 1 [file 40529_2014_9070_MOESM1_ESM.gif]

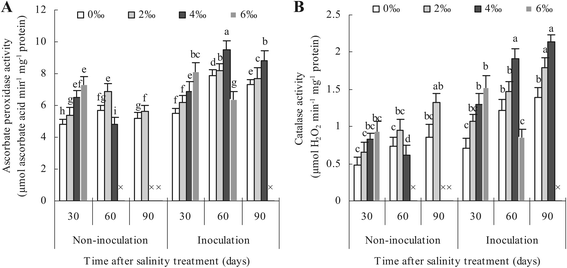

Supplement: Supplementary file 2 — Authors’ original file for figure 2 [file 40529_2014_9070_MOESM2_ESM.gif]

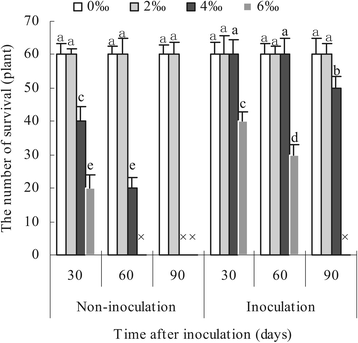

Supplement: Supplementary file 3 — Authors’ original file for figure 3 [file 40529_2014_9070_MOESM3_ESM.gif]
